# Traditional Mapuche ecological knowledge in Patagonia, Argentina: fishes and other living beings inhabiting continental waters, as a reflection of processes of change

**DOI:** 10.1186/s13002-016-0130-y

**Published:** 2016-12-07

**Authors:** Juana Aigo, Ana Ladio

**Affiliations:** 1 IDEAUs (Instituto de Diversidad y Evolución Austral- CCT CONICET- CENPAT), Bv. Alte. Brown 2915, Puerto Madryn, 9120 Chubut Argentina; 2 INIBIOMA (Grupo de Etnobiología. Instituto de Biodiversidad y Medioambiente (INIBIOMA– CONICET), Quintral 1250, San Carlos de Bariloche, 8400 Río Negro Argentina

**Keywords:** Ethnoichthyology, Fluvial environments, Fish, Perception

## Abstract

**Background:**

Understanding how people interpret environmental change and develop practices in response to such change is essential to comprehend human resource use. In the cosmology of the American indigenous peoples, as among the Mapuche people, freshwater systems are considered a living entity, where animals have an enormous role to play in the universe of meaning. However, human adaptive responses to freshwater system dynamics are scarcely examined. In this work a survey is carried out in three Mapuche communities of Argentine Patagonia to assess their traditional knowledge of the fishes and other non-human living beings that inhabit lakes and rivers. Both material and symbolic aspects are included, as are the differences in knowledge and use of the fishes between past and present times.

**Methods:**

Our methods were based on a quali-quantitative fieldwork approach. In-depth interviews were carried out with 36 individuals from three rural Mapuche populations in Neuquén province (Patagonia, Argentina). Free listing was used for inquiring about fish knowledge and use. Fishes were identified scientifically and ethnotaxonomically. In-depth analysis of the discourses was conducted, documenting the recognition, perception, and cultural significance of fluvial environments and their inhabitants. Quantitative survey results were analyzed with categorical statistical methods.

**Results:**

The body of knowledge of the communities studied reflects the socio-environmental changes experienced by Patagonian freshwater bodies. According to local perception, non-human beings live in these water bodies, guarding the environment, and they should not be disturbed. At present, five different fish species are identified, three of which are exotic, having been introduced at the beginning of the 20th century by the white man. These exotic trout (*Oncorhynchus mykiss* and *Salvelinus fontinalis*) are considered ill omens, indicators of the white man’s presence, and therefore their appearance presages negative events for the families. In addition, we found that Mapuche people differentiate fish species mainly by morphological, organoleptic and ecological attributes. Current consumption of fish by Mapuche communities is sporadic, in accordance with bibliography and ancient tales. Several fishing tools are used, including modern elements.

**Conclusions:**

Our data enable us to characterise dynamic traditional knowledge in these communities, which is flexible in nature and adaptable to new situations, demonstrated by the incorporation not only of new species but also new fishing tools. It also seems that new significances become absorbed in synchrony with the advance or arrival of exotic and invasive species. For the Mapuche, the presence of the white man heralded by exotic trouts speaks of how a recent event, such as the introduction of the salmonids, is already incorporated into Mapuche symbolism. Mapuche traditional knowledge and cosmovision on the use of fish and waters, a vision which promotes respect and the avoidance of actions that could disturb the beings (animals and sacred or mythological characters) that inhabit and take care of them should be fostered as part of management plans of regional natural resources. This paper contributes to the broader literature on freshwater resource management by providing empirical evidence of the critical role of local perceptions in promoting the sustainable management of natural resources.

## Background

Throughout history aquatic environments have played a key role not only in the development and survival of local communities, but also in providing habitat and refuge for ichthyofauna [1]. At a global level, fish occupy an important place in fishing communities or those with access to rivers, lakes and seas [2, 3]. In Patagonia, fish currently constitute one of the principal recreational sport and tourist resources [4–6], and a great diversity of ecological and biological studies have been carried out related to both native and exotic fish and the Andean-Patagonian aquatic environments they inhabit [6–9]. Nevertheless, very little is known about how freshwater resources, and in particular fish, are perceived and used by local people, especially the indigenous Mapuche communities.

In their cultural framework the Mapuche people (“people of the land”; *mapu* = land; *che* = people) show great diversity in their ways of communicating and relating to nature, making them specialists in the care and management of natural resources [10]. Both their native language (Mapudungun) and the names of their communities reflect the vital connection between the people and their land [10]. In Patagonia several Mapuche populations and their descendants live in areas around streams and lakes, which are used by the rural inhabitants to cover their basic consumption and personal hygiene needs. Nevertheless, fish resources appear not to be fully used, given that at the end of the 19th century the Mapuche people incorporated livestock breeding as central to their way of life and as an essential means of subsistence [11].

As in the majority of American indigenous peoples’ cosmologies, for Mapuche people all of nature is alive, populated by beings or entities they have social relationships with [12] – relations between humans and nature where the visible and invisible worlds overlap or intersect [13]. For the Mapuche people, animals in particular have enormous relevance in the universe of meanings [14–16] and are strongly connected with the different spheres of their reality. This includes subsistence relationships [17], their connection with forebears and their ancestral lines [18], the representation of their social and ritual organisation [13] and origin myths [19, 20] among others. Thus, animals are part of their oral tradition and therefore subject to processes of cultural transmission [21, 22].

As was stated by Carpenter and Gunderson [23] and Berkes et al. [24], in order to understand natural resource use, it is necessary to study how humans deal with social and ecological change. Many investigations have shown that many native societies around the world have coevolved with resource and ecosystem dynamics and have developed different interpretations, knowledge and practices to live with change and uncertainty [25–27]. Such wisdom tends to evolve into social rules and specific cultural and symbolic dynamics, and supports the emergence of adaptive responses based on their cosmovision [28].

Successful plans for management of fauna and habitat require understanding the value local inhabitants put on the biodiversity of their surroundings, so as to incorporate the significance it has for them [29–31]. Ethnobiology seeks an integral approach that includes people and their needs, transcending mere utilitarian and economic aspects in order to reach an understanding of the cultural value of traditional knowledge [31]. Traditional ecological knowledge has been defined as the body of accumulated knowledge, beliefs and practices developed by local populations through adaptive processes generated through continuous, permanent contact with their environment [28]. From this ethnobiological approach, traditional faunal knowledge (TFK) is the result of the relationship between the cosmos (beliefs, emotions and symbolic representations), the corpus (general environmental knowledge), and the praxis (the behaviours carried out in relation to the use of Nature) [32, 33].

In Patagonia, the study of TFK is scarce if compared with ethnobotanical studies [34–36]. This situation is similar in the rest of the world [29], where the main investigations have focused on aspects related to food and medicine [16, 37–40], symbolic-religious questions [41, 42], environmental and emotional perception [43, 44], and ethnoclassification [18, 45, 46]. Several studies about ethnoichthyology are recorded in South America and Asia which have contributed an invaluable and useful source of new data about the biology and ecology of fish from the point of view of people that interact with and depend on them [2, 3, 45, 47–50]. Ethnozoological studies in Argentina have concentrated mainly on the north of the country, focusing on aspects associated with traditional subsistence, the use of wild birds and fish [15, 42, 51, 52], and the medicinal use of animals [39], as well as studies on traditional management practices of camelids, bees and wasps [53–55]. In Patagonia, the available ethnozoological information is sketchy, focusing primarily on aspects of large mammal hunting (pumas and guanacos) [56, 57], and to a lesser extent, the harvest of molluscs, birds and marine fish mainly by pre-Hispanic societies [57, 58]. Villagrán et al. [18] and Castro [21] have contributed to Mapuche ethnoclassification of some animals and their uses, revealing aspects of their cultural significance, while Herrmann et al. [58] have investigated the symbolic roots of the hunting of wild cats.

For the specific case of freshwater fish and their association with Mapuche people living to the east of the Andes Mountains, it is not easy to reconstruct a clear panorama due to the paucity of specific studies. Ichthyological records for the region do show the use of fish as a food resource (although to a small extent) by hunter-gatherer populations that inhabited the cordillera/pre cordillera and Patagonian steppe [59–63]. However, other authors report only occasional consumption and little cultural value [60], and they coincide with many chroniclers [64–68] as to the scarce interest paid to freshwater fish by the indigenous peoples of continental Patagonia. Exceptions, however, are found in the Mapuche groups on the Chilean side of Patagonia. Dillehay and Navarro [69] have particularly pointed out the importance of the exploitation and use of marine resources in the residence patterns of the Mapuche living on the Pacific coasts. In addition, some ethnohistorical sources (18th century) have indicated that fishing and the use of aquatic environments was important in the past for Mapuche people in the *Araucanía* province of Chile (IX region) [70].

Considering the limited information available on traditional Mapuche knowledge in terms of continental waters and their inhabitants, this work constitutes a first approach to the subject, and in particular to Mapuche ichthyological knowledge in Patagonia. Our analysis of the ichthyological knowledge deals with some aspects of the local perception related to classification as a first step in a more complete interpretation of the body of knowledge about fish. Our approach is not intended to account for the complex Mapuche ethnotaxonomy of fish. Further studies on fish ethnoclassification are required in the future with the construction of a systematized and specific field database that allows an in-depth analysis of this theme in the region.

As a general aim, we proposed the study of Mapuche traditional knowledge of fish and other beings living in the Andean-Patagonian fluvial waters. Basing our work on case studies of three rural Mapuche populations in Neuquén province (Patagonia, Argentina), our specific objectives were: 1) To describe how the water bodies, fish and other beings are perceived culturally, and analyze associated new symbolism in response of changing circumstances; 2) To describe ichthyological knowledge and the use of fish as a food resource; 3) To survey the techniques and tools used for fishing; 4) To identify the differences in current knowledge and practices with those of past times as a form of adaptive capacity.

## Methods

### Study area

**Fig. 1:**
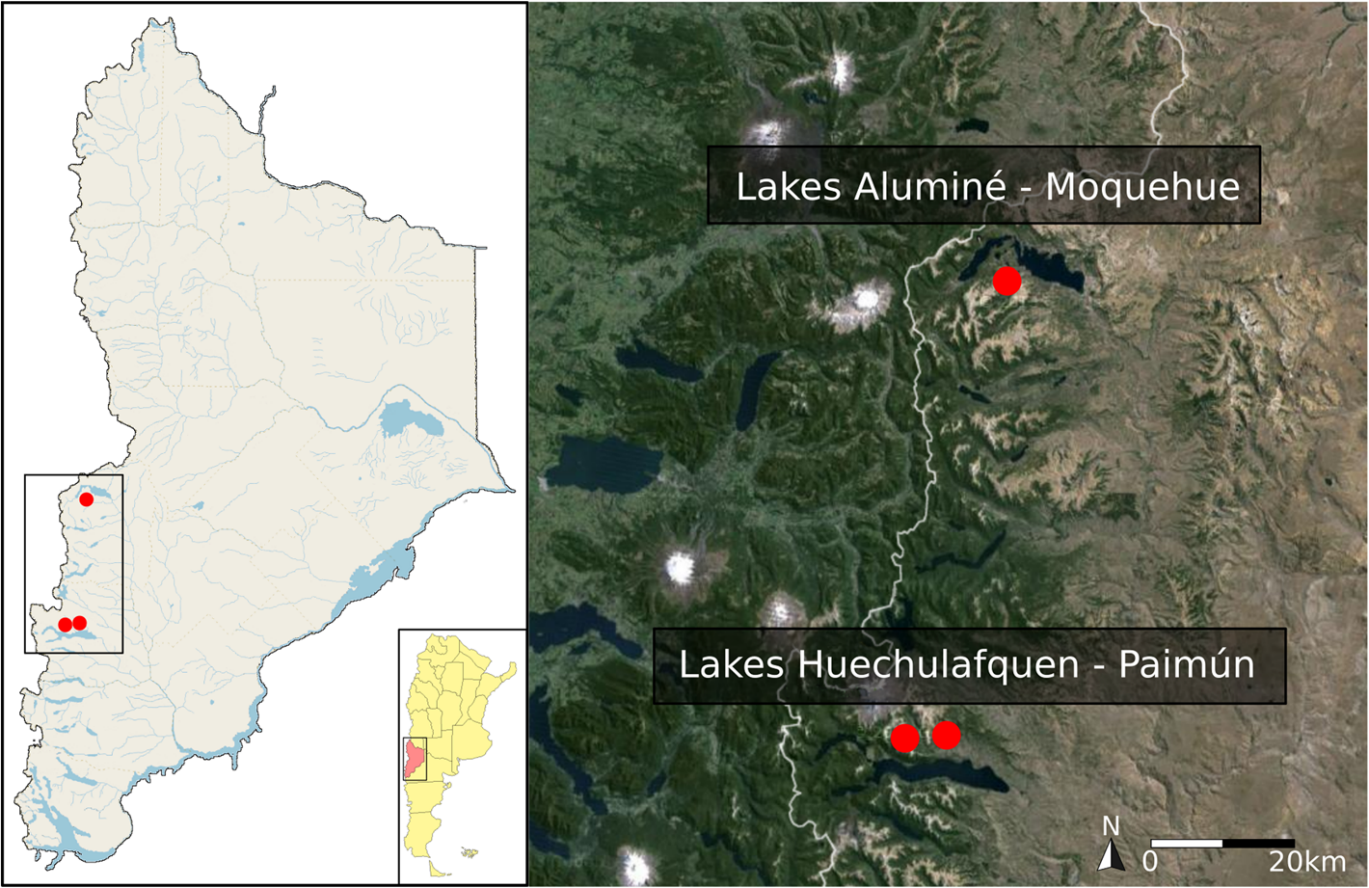
Study area. Location of Mapuche Communities in the Province of Neuquén, Patagonia, Argentina. Community Puel (Lake Aluminé-Moquehue) and Communities Raquithué and Lafquenche (Lake Huechulafquen- Paimún)

The study was carried out in an area of Patagonia east of the Andes, in the Neuquén province of Argentina (Fig. 1). Within this area, Lagos Aluminé, Huechulafquen and Paimún, within the phytogeographical region known as Bosque Andino Patagónico or Sub-Antarctic Forest, are headwater lakes of the Limay River watershed. Lakes Huechulafquen and Paimún are located within Lanín National Park (LNP, 39° 7′, 40° 40′ S, 71° 42′, 71° 12′ W), created in 1937 (Fig. 1). All three lakes are part of the so called Lakes Corridor, a growing tourist destination in Patagonia [71].

Local people who have inhabited this area since ancient times belong to the Mapuche communities of Puel, Raquithué and Lafquenche. The populations making up these communities are small, with no more than 50 families each, and are mainly situated on the coast of water bodies, in intimate contact with these environments (Fig. 1). The Raquithué and Lafquenche communities live on the northern coast of lakes Huechulafquen and Paimún, while the Puel community is situated on the edge of a lake system called Angostura Sur, on the southern coast of Lake Aluminé.

The communities Raquithué and Lafquenche are close together (approximately 2 km apart), at a distance of 25 km from the city of Junín de los Andes (16.510 inhabitants; [72]). Puel is close to the tourist village of Villa Pehuenia (700 inhabitants; [72]) and through this village is linked to Junín de los Andes and other towns or tourist destinations which form part of the previously mentioned Lakes Corridor (Fig. 1). Core economic activities of these Mapuche communities are subsistence horticulture and extensive livestock breeding, but nowadays diverse services related to tourism such as campsites and horse rental, or the sale of handicrafts are becoming the most important economic activities.

The Mapuche people have suffered instances of severe genocide and expulsion since the so called “Desert Campaign” [73] and also as a consequence of the creation of the National Parks system. In particular, the creation of Lanín National Park in 1937 generated profound social and environmental conflict with the Mapuche communities. Most were expelled from their traditional lands, but a few families managed to stay within the park on promising to pay occupation and grazing licences, in addition to accepting limits on their livestock numbers and extraction of forest resources [74–76]. At the present time the indigenous communities and National Parks administration carry out a co-management policy [76, 77], and are experiencing a strong process of territorial and cultural vindication [78–80].

The flora characteristic of the area corresponds to the Sub-Antarctic forest, including a variety of native forest species among which monkey puzzle/pehuén (*Araucaria araucana*), southern beech/raulí (*Nothofagus nervosa*) and the roble beech/roble pellín (*Nothofagus obliqua*) [81] stand out, the pehuén constituting a food resource of high cultural value [77]. Extensive plantations of exotic pines (*Pinus* sp) dominate some areas, such as parts of the coast of Lake Aluminé. The climate is cold temperate and wet, with snow during winter. Rainfall, which is abundant, diminishes from west to east. Average temperatures are 4 °C in winter and 20 °C in summer. The fauna is composed of 166 native vertebrate species, with birds being the most species diverse, followed by mammals, reptiles, fish and finally, amphibians [82].

Among the native fish species present in Patagonian aquatic environments, those which stand out are the perca criolla (*Percichthys trucha*), pejerrey patagónico (*Odonthestes hatcheri*), puyén chico (*Galaxias maculatus*), puyén grande (*Galaxias platei*), bagre aterciopelado (*Diplomystes viedmensis*) and bagrecito del torrente (*Hatcheria macraei*) [9]. Exotic salmonids predominate, such as the rainbow trout (*Oncorhynchus mykiss*), brown trout (*Salmo trutta*), brook trout (*Salvelinus fontinalis*) and Atlantic salmon (*Salmo salar*). Salmonids were introduced in Andean-Patagonian rivers and lakes at the beginning of the 20th century, as part of a national government programme, maintained later by private efforts [83].

### Collection of ethnozoological data

#### Field study

In this study a quali-quantitative approach was used, combined with ethnographic fieldwork and bibliographical reviews. Fieldwork was carried out within the framework of the principles established by the Convention on Biological Diversity (CBD, 1992), during spring-summer of 2011–2012. Authorization for this study was requested from the local leader (“*lonco*” in the Mapudungun language) and informed consent was requested from each informant. Data on fluvial environments and fish were obtained by participant observation, free listing and in-depth and semi-structured interviews. Interviews selection was made through a snowball sampling technique [84], starting with the identification and selection of key primary informants who held more specific knowledge about the topic. At the end of each interview, informants were asked to give the name(s) of other persons who had experience and knowledge in relation to the water bodies and fishes. Using this method, 36 families in total were interviewed. All communities share the same cultural heritage, with strong family ties; in Puel 12 people were interviewed (4 women and 8 men, average age X = 31 ± 22); in Raquithué, 13 people (4 women and 9 men, X = 54 ± 20) and in Lafquenche 7 people (4 women and 3 men, X = 68 ± 13). Free listing was used particularly for inquiring about fish knowledge and use, as well as Mapudungun names [85]. In-depth analysis of the discourses was also conducted, documenting the perception, recognition and cultural significance of these fluvial environments and their inhabitants, as well as the cosmos-corpus-praxis approach [86].

An open interview was first carried out, with broad, general questions, followed by a questionnaire with more specific questions, as a way of orienting the interview, with deeper questions on: 1) the cultural, emotional and symbolic perception associated with the water bodies, fish and other beings. 2) ichthyological knowledge and the use of fish as a food resource (common names of the fish which are known and used, their uses, their morphological characteristics); 3) the techniques and tools used for fishing, and 4) the informant’s perception of the differences in current knowledge and practices compared with past times (their grandparents’, great-grandparents’ and great-great-grandparents’ generations, from the third to the fifth generation of informant). In addition, attention was focused on obtaining traditional Mapuche tales or stories (”*Epew*” in Mapudungun) or personal experiences recounted by locals. In some cases walks with informants were also carried out [86]. Fish identification was carried out in the field from direct observation of specimens when possible and/or photographic material of the fish species. This material was presented to informants, and they were asked to describe the fish [45].

### Data analysis

Given the varied nature of the information in terms of the kind of data and its different epistemological approach, data analysis was mainly descriptive-interpretative [77, 85]. Total species richness of fishes was estimated using the total sum of species. Inhabitants’ answers were analysed systematically, recording agreement and consensus between informants’ discourse fragments [45]. The consensus of answers (% C) was calculated by totalling the number of informants who cited an item in each case in relation to the total number of informants (n/N × 100) [87]. The data obtained were analysed using an emic/etic approach, consisting of comparison between indigenous knowledge (emic) and academic knowledge. Ethnolinguistic information was analysed by linguists from the community (Gabriel Cañicull, Raquithué community) and the help of Mapudungun dictionaries [88]. To improve interpretation, etic categories were drawn up to group for example, the natural beings of pre-Hispanic and post-Hispanic origin and mythical beings, sacred and/or religious. In the same way, emotional perception of the water bodies was analysed, grouping informants’ expressions in 6 general categories [43]. Local criteria for describing the different fish species were also analysed, following the methodology used by Molares and Ladio [36], grouping characteristics of an organoleptic and ecological nature directly from the discourses, which are presented verbatim in Tables 2 and 3. Fishes were identified (nomenclature and biogeographical origin) using the dichotomous keys and field guides for Patagonian fishes and continental environments in Argentina [89–93] and revised by Dr. J. Aigo (Instituto de Diversidad y Evolucion Austral, CONICET). Quantitative information obtained from the interviews was analysed by means of statistical comparison of categories using the Binomial Test (*p* < 0.05). The % C of native and exotic species was compared with Mann–Whitney (*p* < 0.05).

## Results and discussion

### Cultural, emotional and symbolic perception of fluvial environments

#### Guardians or owners of the water

The Mapuche people perceive Nature as being animated, as do most American indigenous peoples, and the inhabitants of the three communities visited during this study believe in the existence of forces, guardians, protectors or owners of nature who are in charge of the care, protection or preservation of the different natural resources. The Mapuche inhabitants perceive the water (“*ko*”) and the lakes or lagoons (“*lafquen*”) as living entities, with a soul (“*allhu*e”) and specific force, or owner (“*nwen*”) which must be respected, left undisturbed and from whom permission should be requested if one wants to enter. In other cultures where traditional hunting and fishing form part of the subsistence way of life, it has been observed that one of the most notable aspects of the cosmological conception is the presence of sacred or mythological beings and/or animals considered “protectors or owners” of the fauna, from whom permission must be granted before any removal [94, 95]. Our field data record the presence of the *“nwenko”* or the owner of the water, in accordance with records from literary sources and/or published studies [13, 20, 70]. For example, Bengoa [70] refers to the “*nwenko*” or owner of the water by the name “*Chompallhue*”. According to Kuramochi [20] and Mora Penroz [13], on the other hand, the “*nwenko*” is recognized as “*Shumpall*”, a generic name for the personification of the waters. All the other water beings, therefore, are forms taken by the “*Shumpall*”, which can represent a man, woman, sheep, hide, bull, pig, horse, or stone [20].

**Fig. 2:**
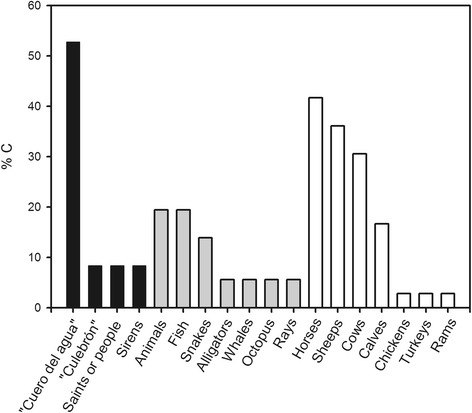
Categories of ‘water beings’ mentioned by the inhabitants of Mapuche Communities and percentage consensus (%C) in the 36 interviews conducted in the study area. Post-Hispanic animals, N 7 (*black bars*), pre-Hispanic animals, N 7 (*grey bars*) and mythical beings, N 4 (*white bars*)

Similarly, in this work, by means of a compilation of inhabitants’ stories, the presence of the “*nwenko*” was found in association with 18 different categories, 14 of which correspond to water beings, generally represented by animals (Fig. 2), and 4 by sacred and/or religious mythical beings, such as the “*cuero del agua*”, the “*culebrón*”, the “*sirena*” and the “*santos*” (water hide, snake, siren, saints) (Fig. 2). In order of importance, the main personage cited was the “*cuero del agua*” or in the Mapudungun language, “*Trelquehuecufe* or *Trelwekufe*”, according to the bibliography [13, 70]. In addition, our results enable us to distinguish among the guardians of the water numerous types of animals which are post-Hispanic in origin (7 in total), that is, animals that arrived in the region through contact with Europeans (sheep, horses, cows, bulls or calves, turkeys, rams and hens), and which are strongly associated with everyday domestic and farm work. The post-Hispanic animals such as the horse, cow, sheep and calves, according to the number of cites, occupy second place in terms of cultural importance, with fish following in third place (Fig. 2). Interestingly, in the current discourses we see comparatively more domestic, post-Hispanic animals associated with water bodies than sacred or religious mythical elements. At the same time, the frequency of beings that are post-Hispanic in origin (66%) is greater than the pre-Hispanic elements, such as fish, whales, octopuses, rays, crocodiles and snakes (34%) (Binomial Test, *p* < 0.05). In particular, in comparison with bibliographical sources [13] we find that the “*Shumpall*”, assumes a wider repertoire, including new animals not mentioned in previous studies, mainly domestic animals.

Our results allow us to see, on the one hand, the hybridisation of post-Hispanic elements of Nature, such as animals adopted after the Spanish conquest, and on the other hand the incorporation of Christian elements such as the presence of the “*saints*”, which indicates the adoption of religious syncretism by Mapuche inhabitants (Fig. 2). This process of cultural hybridisation, which enriches the vision of the different cultures in relation to their surroundings with new symbolic and religious elements [96], reveals the dynamic processes involved in the construction of knowledge and their symbolic interpretation [34], and the influence of socio-environmental changes (and therefore of the species present in these surroundings) in the preponderance of certain types of knowledge over others [44]. This particular perception forms part of a network where dialectical relationships at several different levels – natural, non-natural and social – are interwoven in these communities. The dialogue that takes place between these inhabitants and their territory (aquatic and non-aquatic), the climate, animals and plants is fundamental [97]. Consequently, the current co-dependence on domestic livestock as the principle way of life appears to be reflected in their mythical world, where sheep and cows take on an important role as guardians of the water. In this way, in accordance with Ladio and Albuquerque [96], knowledge is transformed, and this transformed logic is what keeps traditional ecological knowledge alive in many indigenous communities.

#### The emotional relationship between the Mapuche people and their fluvial environments

Our records enable us to witness the special relationship with fluvial aquatic environments that exists within the Mapuche cosmovision. In this work the inhabitants interviewed spoke of a strong link with the water and its associated beings, which is reflected in the different attitudes and emotions aroused towards them, which were very diverse. Of all the informants, the feelings mainly observed towards the water were that of “*respect”* for this resource (100% of interviewees). Inhabitants expressed the importance of transmitting this “*respect*” for the water bodies to both the new generations and tourists. “*Respect*” refers to being able to visit their communal sites without loud noises or any other disruption, otherwise the waters may become angry. The notion of “*respect*” predominates in many traditional belief systems as an ethical imperative that affirms that it is necessary to preserve biodiversity to ensure ecosystem processes in the long term [16, 98, 99]. Several authors [100–104] have noted in different societies, the intimate relation that exists between the notion of “*respect*” and the construction of cultural believes, such as social taboos, that encodes distinct rules, norms and sanctions to promote natural resources sustainability. In the particular case of freshwater systems and their fishes, which in general are common-pool resources, sustainability in indigenous and rural communities could only be possible by such norms of behavior, conventions and self-imposed codes of conduct that guide the society actions [99, 105, 106], which collectively are refer to as informal institutions [107–109].

Several studies in rural communities of Brazilian cost and the Amazon have shown that most of the harvesting and management actions that they undertake are, at least partially, shaped by their local perceptions and taboos regarding fish consumption [2, 37, 110, 111]. In addition, in the Andean literature, a set of sociocultural rules of behaviour associated to the Quichua cultural tradition and the lagoons called “*Cochas*” have been described [112]. Like the Patagonian freshwater environments, the “*Cochas*” are inhabited by enchanted beings. Similarly, the Andean “*Cochas*” foster actions, attitudes and care practices that reflect their material, conceptual and energetic configuration with the “*Pacha Mama*” [112].

In our case, 56% of individuals interviewed expressed attitudes of “*care”* associated with actions of environmental protection and self-protection in relation to the water body. Informants indicated that if these actions are carried out, greater collective wellbeing is generated, and suffering from accidents, illnesses or deformities can be prevented. In this sense, the interwoven co-dependence with domestic animals is again evident in the dialectics, since these illnesses or deformities may occur either in the people themselves or in the livestock, if not taken care of. In addition, cited simultaneously with this attitude of “*care*”, is the feeling of danger, risk or threat, manifested through “*fear*” (42%). Inhabitants claim that the force of the “*nwenko*” will react negatively towards people who remove fish by force, or use more force than necessary, and therefore locals are careful not to carry out or participate in incorrect actions performed on the water bodies.

The emotional and attitudinal significance refers to relationships that should be interpreted as parts of a whole. The “*respect*”, “*care*” and “*fear*” are feelings which have been interpreted by several authors as the most eloquent forms of ethno-conservationism, where some practices in the use of the environment by traditional communities are in synchrony with a preserving attitude, due to the supreme character of the land in their cosmovision [113, 114]. In this sense, the “*respect*” for aquatic environments and their guardian beings described by informants cannot be isolated from the Mapuche cultural context where “*respect*” for “*Mapu”* (the land) constitutes the basic foundation of their identity. Furthermore, water as an element of nature is perceived to be associated with “*luck”* (6%); water and its protective beings are considered as having strength enough to determine that certain events or circumstances may result in a specific outcome. For example, some inhabitants said they believed that the action of pulling out the hair of a ram, one of the owners of the water, can bring either good or bad luck, depending on the occasion. Apparitions and exchanges with mythical beings are recurrent in American mythological literature as situations which modify the destiny of participants [115]. Finally, with fewer cites, interviewees mentioned values or utilitarian-type relationships through the perception of water as a “*medicine*” (8%; “*medicina* or *Lahuen”* in Mapudungun). Within the Mapuche culture, water is seen as a significant vehicle for the transmission of health, highlighting for example the importance of the daily practice of morning bathing as a “*medicine*” [36, 116] and the consumption and access to pure water for the preparation of food and to satisfy thirst. Furthermore, the use of medicinal plants, a fundamental part of Traditional Medicine, is practised mainly in the form of infusions and watery decoctions or drinks, baths, poultices, and syrups, among others, and depends fundamentally on the availability of safe, fresh water [36].

Finally, some informants related water to “*romantic conquests”* (6%), in the sense of the power exercised by mythical beings to win over men and women. The water beings will try to have romantic conquests with the people; in the case of women, the “*water hide*” is talked about and for men the “*siren*”. This shows once again the importance of the symbolism of water bodies and their beings in the emotional and sentimental lives of people. Similarly, in Chilote mythology an example is given that refers to the romantic conquest of the mythological figure of the “*Trauco”* who inhabits the forests, a character who will seduce women, overcoming their willpower. The aquatic mythological beings within the Mapuche culture are very similar to those found in Chilote mythology in association with marine environments [117]. However, in both cases the existence of a mythology of their own is evident, formed through syncretic processes acting on cultural and religious elements from colonial times, and from marine explorers [117].

The results mentioned above show how the social relationships between humans and nature interweave the visible world (cow, horse, sheep) with the invisible one (water hide, snake, siren, saints). According to Mora Penroz [13], the use of resources within the Mapuche cosmovision depends strongly on the relationship between these two worlds (visible and invisible). The spirit world stands out, related to sacred sites and places, plants and animals which co-inhabit the land and form part of this social and spiritual universe [118]. In agreement with Molares and Ladio [119], the evidence to support our work allows us to establish that interviewees speak of elements belonging to a relational kind of cosmovision, where Nature is considered a living force. This cosmovision sustains a way of life where elements in the surroundings, such as water, have a soul and should be respected; humans are not superior to them, but submit to the designs of a system in which human and non-human beings participate equally. This cosmovision currently lives alongside and confronts other, anthropocentric perspectives, such as those of western consumer societies, where natural resources are understood as plausible elements for human exploitation and subjection [120]. In this sense, the cultivation of exotic fish in different fluvial environments with the objective of economic exploitation alludes to this anthropocentric vision which has been promoted by the Argentine government for a long time now.

Our results are also in line with other studies carried out in Latin America on environmental perception in traditional communities (i.e. [121–123]) where attitudes shown towards the environment and its components, as in the case of animals, appear to be associated with ideological variables which imply acceptance and observation of cultural norms and rules related to the regulation and sustainable use of natural resources. Our results therefore contribute new perspectives and accentuate the value of a model for the management and use of aquatic environments and their fauna which emerges from the traditional knowledge of the Mapuche people – a management model with a strong spiritual base [10], but which has not been visible up to now.

### Traditional knowledge of fish, and their use as a food resource

#### The group of fish and their local names

In the three communities studied, fish are known generically as “*Challwa*” in the Mapudungun language, as found by Villagrán et al. [18]. This name in the native language constitutes an onomatopoeic word (an onomatopoeic name) alluding to the sound produced by fish in the water as they move or swim (Ayllapan pers. com.). According to the principles of categorization and nomenclature proposed by Berlin [124] and those used by Villagrán et al. [18], the name “*Challwa*” represents an ethnocategory corresponding to the grouping or class Fish, distinguished by their appearance or general morphology. Other examples of Mapuche names expressed in the same way are “*kullin”* (mammals), “*üñüm”* (birds) and “*fillkuñ”* (lizards), all of which correspond to Class “life form”. This generic ethnocategory related to the general morphology of the body used by local people is the most commonly recorded in the folk classification systems studied in fish works mostly for South America [2, 37, 45, 125].

In our work specifically, 63% of informants referred to fish by the name “*Challwa*”, while the remaining 37% used the generic name “*peces*” (fishes in Spanish) and said they did not know the names in the native language. We did not distinguish ethnocategories, unlike Villagrán et al. [18], who found hierarchically subordinate species within the class Fishes, and registered names with particular etymologies of great ethnographical richness.

**Fig. 3:**
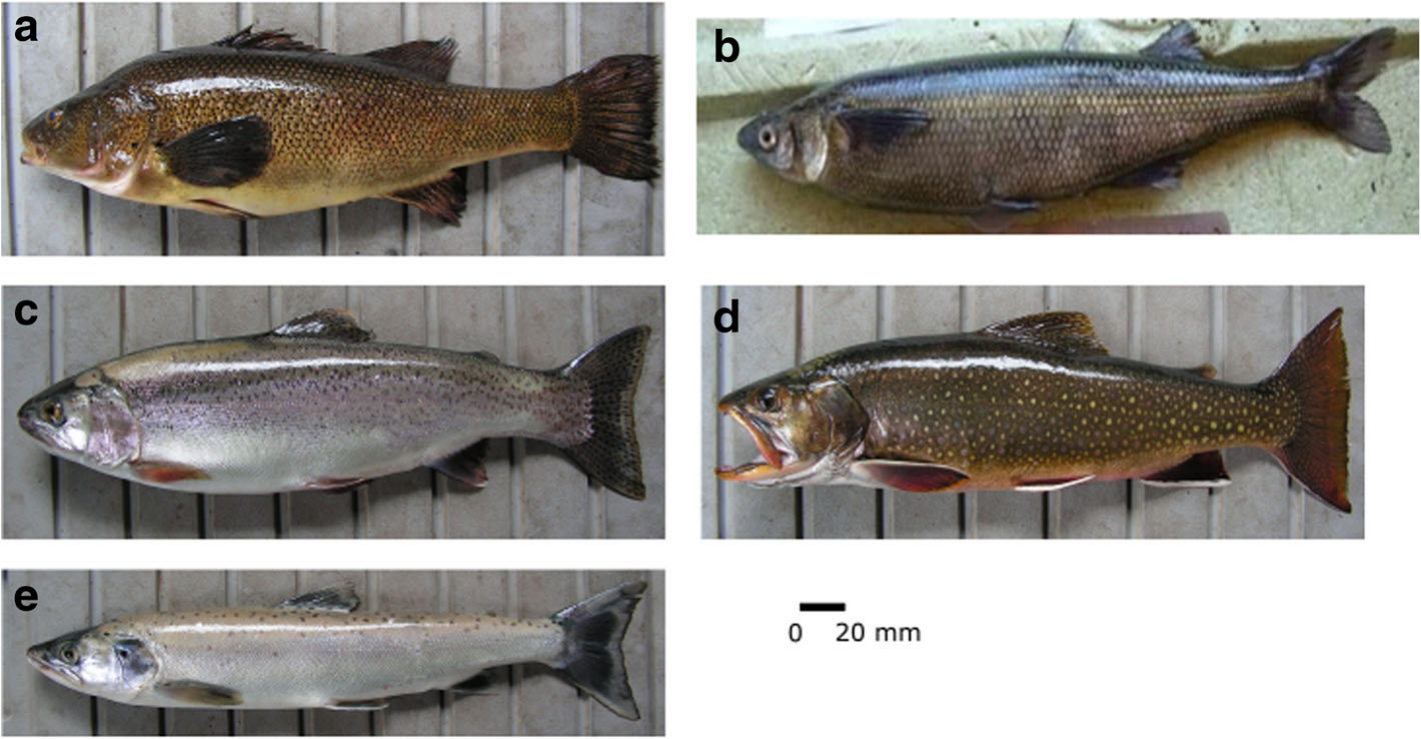
Main fish species known and consumed in Mapuche communities. **a** Perca o trucha criolla, *Percichthys trucha*; **b** Pejerrey patagónico, *Odontesthes hatchery;* **c** Rainbow trout, *Oncorhynchus mykiss;* **d** Brook trout, *Salvelinus fontinalis* and **e** Atlantic salmon, *Salmo salar.* Photos: Juana Aigo (Laboratorio de Ictiologia y Acuicultura experimental, INIBIOMA)

In our work a total of five fish species were recognized by the people, of which two were native species, perca criolla (*Percichthys trucha*) and pejerrey patagónico (*Odontesthes hatcheri*), and three were exotic, rainbow trout (*Oncorhynchus mykiss*), brook trout (*Salvelinus fontinalis*) and Atlantic salmon (*Salmo salar*) (Table 1, Fig. 3). It should be pointed out that at a regional level, in the Limay river basin which the studied environments belong to, other native (*Diplomystes viedmensis*, *H. macraei*, *Galaxias maculatus* and *G. platei*), and exotic (*S. trutta*) species are also present but were not cited by informants. Nevertheless, the species cited here show consistency with the local fauna of each environment, and also with respect to the unequal impact of salmonids observed in rivers and lakes [126, 127]. It is known that the salmonids, and *O. mykiss* in particular, appear to displace the native fish almost completely, and nowadays captures of *H. macraei*, *D. viedmensis*, *G. maculatus* or *P. trucha* are rare, especially in rivers [9, 128]. Moreover, according to ecological investigations, the species cited are the most widely distributed and currently most abundant in the region [9]. In Table 1 the principal characteristics of the species and their most frequent habitats are described.

**Table 1:** Fish species known by inhabitants. Local name, scientific name, order and family, habitat, distribution, origin and percentage consumption (%C) of each species in relation to the total no. of interviewees (*n* = 36) are given

Local name	Scientific name	Order and family	Habitat	Distribution	Origin	(%)C
Perca/ Trucha criolla	*Percichthys trucha*	Perciformes, Percichthyidae	Fresh and/or brackish water Benthic habitat	Endemic to Argentina. Patagonian rivers and lakes in central and southern Argentina and Chile.	*Native*	37
Rainbow trout	*Oncorhynchus mykiss*	Salmoniformes, Salmonidae	Freshwater; marine; brackish. Benthic-pelagic habitat; Anadromous.	Native of northern hemisphere. Extensively introduced in cold waters in different parts of North America and the rest of the world.	*Exotic*	30
Brook trout	*Salvelinus fontinalis*	Salmoniformes, Salmonidae	Freshwater; marine; brackish. Benthic-pelagic habitat; Anadromous.	Native of northern hemisphere. Extensively introduced in cold waters in different parts of North America and the rest of the world.	*Exotic*	15
Pejerrey patagónico	*Odontesthes hatcheri*	Atheriniformes, Atherinopsidae	Freshwater, cold-temperate Benthic-pelagic habitat.	Endemic to Argentina. Patagonian rivers and lakes in Argentina and Chile.	*Native*	13
Atlantic Salmon	*Salmo salar*	Salmoniformes, Salmonidae	Freshwater; marine; brackish. Benthic-pelagic habitat; Anadromous.	Native of northern hemisphere. Extensively introduced in temperate Arctic and cold waters in different parts of North America, Europe, New Zealand, Australia, Chile and southern Argentina.	*Exotic*	5

#### Local interpretations and descriptors of the fish

In the species descriptions provided by the people interviewed we were able to identify two types of descriptors or classification criteria, one which was organoleptic in nature and the other ecological (Table 2). Within the organoleptic descriptors we included attributes such as size, shape, colour and taste. Within the ecological group were attributes referring to abundance, habitat, behavior and whether or not the species had been introduced by man. The classification criteria recorded in this work in relation to morphology, habitat, behavior as well as the morphological analogy with other animals coincide with those described by Villagrán et al. [18], regarding Mapuche ethnoclassification and with those in different ethnotaxonomic studies of fish from South America such as Begossi and Garavello [129], Marques [45], Mourão and Nordi [46, 130] and Pinto et al. [125].

**Table 2:** Classification criteria, descriptor attributes, and qualities used by inhabitants to identify the fish species

Criteria	Descriptor attributes	Descriptor qualities in the discourse
Organoleptic	General body size	Large body Small body
	General body shape	Wide body Rounded body
	Body structure	Has spines Has a lot of bones
	Body colour	Red White Blue Red-brown Silvery brown Has red and yellow specks
	Colour of flesh	White Reddish
	Taste of flesh	Tasty Tasteless Oily
Ecological	Abundance	Many individuals
	Habitat	River
	Behaviour	Invades and kills
	Introductions	“*Cosa de winka*” (thing brought by the white man)

Perca, rainbow, and brook trout are distinguished by organoleptic attributes such as structure, size, shape, taste, and the colour of the body and flesh, which indicates that these species are consumed. The perca is considered a tasteless, oily fish, with many bones, round shape, small size, brown skin and white flesh (Table 3). The morphological analogy with other animals was highlighted, (e.g. “the perca has white meat like the chicken”; Interviewee # 2). In contrast, trout are perceived as tasty, large, and with colourful body (red/brown body colour, presence of specks) and red flesh (Table 3). Trout were also distinguished by some people for their ecological characteristics. They are recognised as river fish that invade and kill other fish and are associated with environmental change. The pejerrey patagónico and salmon, on the other hand, are perceived in a similar way by inhabitants, sharing the same descriptive qualities. This reflects the difficulty commonly found when it comes to differentiating and studying these species, given the similarity of their taxonomic characteristics (Table 3).

**Table 3:** Identification of fish species according to the descriptor qualities used by inhabitants

Name	Descriptor qualities	Inhabitants’ descriptions	Bibliographical description^a^
Perca/ Trucha criolla	Tasteless^(O)^ Oily^(O)^ Ugly^(O)^ Has spines^(O)^ Brown Body^(O)^ Small body ^(O)^ Rounded body^(O)^ Has a lot of bones^(O)^ White flesh^(O)^	“They are the ones with the spine and white flesh …” “The perch has a lot of bones, a lot of spines…” “The colour of the flesh is really white like chicken…” “The perch is tasteless…” “They are almost round fish …” “The perch was always small…”	Principal characteristics: Oblong elongated body. Rather small head. Mouth not very large, including jaw. Small maxilla. Dorsal fin partly spiny and partly soft, separated by a groove. Operculum with small spines. Colouring: Back of the head violet-brown. Scales have coffee-coloured marks or specks. Caudal fin lemon-violet with marks which are almost black. Dimensions: Reaches 350 mm in length and 3.5 kg in weight.
Pejerrey patagónico	Elongated body^(O)^ Silvery brown body^O)^		Principal characteristics: Slender, hydrodynamic body. Narrow caudal peduncle. Colouring: General body colour silvery yellow. Outer edge of each scale has black dots, giving a dark hue.
Rainbow trout	Tasty^(O)^ Nice^(O)^ Red and yellow specks on body^(O)^ Large body^(O)^ Reddish flesh^(O)^ Invades and kills^(E)^ River^(E)^ *“Cosa de winka”* thing of the white man^(E)^	“The ones my dad brought were always big…” “The big fish were trout …” “The trout have reddish flesh …” “It was a fish with red and yellow specks …” “That’s the one that invades and kills the small trout …” “Then there are those red ones, they’ll be the river ones, then there’s the white one, and the reddish brown one…” “The ones my dad brought were always wide…” “That’s the one that invades and kills the other fish…”	Principal characteristics: Large size and rounded belly, body covered in numerous small scales. Has an adipose fin behind the dorsal fin. Caudal fin straight or slightly concave. Large mouth with conical teeth. Colouring: Dark back with olive green reflections, with black spots extending to flanks. A longitudinal purple stripe runs from the eye to the caudal fin, particularly notable in mature specimens. Spotted dorsal and caudal fins. Silver forms exist. Dimensions: 650 mm in length and up to12 kg in weight. Observations: Species of sports value introduced into the country from the USA at the beginning of the 20th century. Some populations live their entire live in lakes, rivers and streams.
Brook trout	Tasty^(O)^ Nice^(O)^ Red-brown body^(O)^ Large body^(O)^ Reddish flesh^(O)^ Invades and kills^(E)^ River^(E)^ *“Cosa de winka”* thing of the white man ^(E)^	“The big fish were trout …” “The trout have reddish flesh …” “The reddish brown one with specks is the tastiest…” “That’s the one that invades and kills the small trout …” “Then there are those red ones, they’ll be the river ones, then there’s the white one…”	Principal characteristics: Differs from the other salmonid species mainly in its characteristic colouring. Colouring: Olive brown with iridescent tones. Back marbled, with vermiculation which extends to dorsal fins. Flanks have red dots surrounded by blue halos and yellow-green spots. Red or orange belly. Dimensions: up to 530 mm in length and up to 12 kg in weight. Observations: Originally from the north east of North America. Introduced into Argentina in 1904 in Rio Negro province. Used for repopulation in pisciculture. Lives in Patagonian rivers and lakes.
Atlantic salmon	Elongated body^(O)^ Silvery brown body^(O)^		Principal characteristics: Large in size. General body shape slender with edge of caudal fin slightly concave. Colouring: Body silvery with bluish head and back. Black spots on flanks, some ‘x’ shaped.

^a^Miquelarena et al. [93]; Del Valle and Nuñez [90]; Wegrzyn and Ortubay [91]

In agreement with Pinto et al. [125] our findings show that criteria for the classification of living beings used by local people or fishermen transcend simple morphology, as proposed by Berlin [124], Hunn [131] and Brown [132]; characters related to appearance, albeit very important, are not the only standards for classifying living beings. Our results are in agreement with diverse studies showing that biophysical and sensorial characteristics of animals are interpreted and classified according to cultural parameters of special meaning and salience to each society, such as those which have emotional connotation [43, 124]. This view is based on the intellectualistic theoretical model that refers the universal tendency of human beings to organize, classify and name the world around them, according to their worldview [124].

Coincidentally, ethnoclassification and perception studies of medicinal plants in Patagonian Mapuche communities found that the classificatory universe was guided primarily by organoleptic characteristics, both morphological and perceptual, such as smell, taste, shape and colour, as well as ecological-environmental characteristics [36]. This similarity speaks of common recognition processes, a subject which should be studied in more depth, considering the coincidences in ways of culturally interpreting the organisms that inhabit the Patagonian region. Finally, it is worthy of mention that, independently of the symbolism and negative ecological aspects associated to exotic trout, and considering the previously mentioned aspects, they are regarded by people interviewed as nice fish, whereas perca are considered ugly fish.

#### Fish consumption

With respect to the consumption of fish by local families, the species with highest percentages of consumption were perca, the rainbow trout and brook trout (Table 1). These species also present a larger number of organoleptic descriptors compared to the pejerrey and salmon (Table 3). The use of fish as food has been reported as one of the main reasons that determines given species to be more widely known than others [125].

Informants commented that in the old days the fish were eaten cooked and that generally the Mapuche people cooked them using embers or hot ash. They also said that on certain occasions the fish were eaten boiled or as “*charqui*”, particularly when travelling for extended time. We know that charqui forms part of their traditional gastronomy, and that a lot of raw food of animal origin used to be consumed [67, 133]. In the case of fish, we found no records of their being eaten raw.

At the present time, the consumption of native and exotic fish species is similar in statistical terms (Mann Whitney Test, *p* = 0.564, Table 1), although the % C of natives (25 ± 17) is slightly superior to exotics (17 ± 13). In this work informants did not cite consumption of fish on special occasions such as celebrations, ceremonies or important meetings. The principal subsistence activity for Mapuche families in the region is sheep, cattle and/or goat breeding, and mutton is the main food, although equally important are the collection of “*piñones”* (the seeds of the *Araucaria araucana*) and the growing of vegetables and fruit in home gardens, for family consumption [134]. It could be said that, at least for the families settled around the aquatic environments studied here, the consumption of fish is not a daily occurrence, but rather sporadic. This has also been cited by Prates [57].

Local knowledge of fish species is found to be associated with their consumption and recreation practices, such as fishing, in the different aquatic environments. The best-known species of fish coincide with those which are most frequently consumed and caught by sports fishermen in the region [90, 91]. Everything seen so far in this study reflects how the traditional ecological knowledge held by inhabitants is synchronised with signs of change in the environments (or landscape), given that we observed not only considerable knowledge of the most important native fish, but also of the exotic species introduced for sporting purposes into the Patagonian lakes and rivers. The salmonids represent a complex problem in that they have a positive impact on regional economy, but generate substantial negative effects in the environment [5–7]. The rainbow trout in particular, despite being a species of great sport value in Patagonia and the most commonly used species in commercial aquaculture [90], has generated significant negative effects in the population of native fishes [7, 135, 136].

#### Symbols and rules in the cultural interpretation of fish

The body of fish knowledge in the communities studied includes wisdom and practices moulded throughout history, which have strong symbolic significance. This is reflected in the tales of the inhabitants, for example, with respect to their knowledge of trout (rainbow and brook) and their perception of them as species that invade and kill other fish present in the aquatic environments. These species which are strongly associated with the presence of the “white man” are given the pejorative name in Mapudungun of “*cosa de winka*” (“white man stuff”), and used to symbolically represent a negative omen. One informant from the Puel community told us *“my grandmother used to tell us….suddenly these big fish appeared, big heads in the river … my grandfather would say, the white men have been close by…”*(Interviewee # 12)*.* This kind of tale constitutes an example of the ominous value of this species, also known locally in rural areas as a “*bad sign*” [10]. In this case, for the Mapuche the arrival of the white man heralded by this fish speaks of how a recent event, such as the introduction of the salmonids, is already incorporated into Mapuche symbolism. This gives us an idea of the negative environmental change that took place due to the invasion of these species and the sociocultural change that goes hand in hand with the arrival of the white man. According to Fernández Bravo [115] this kind of symbolic production provides the community with explanations and didactic elements to use in speaking about significant events in their culture. In Latin American literature we find numerous examples of animals which give “*signs*” of future happenings of a social nature, or animals known as being “*of ill omen*” [137], as is the case of black cats [29]. In his book on Mapuche secrets and legends, Calvo [138] writes that eating fish will bring bad luck.

Specific taboos about fishes have also been described for the Brazilian coast and the Amazon based on perceived toxicity [37, 111], religious symbolism [139, 140], representation of human reincarnation [100], and appearance or behavior [37, 111].

In the Mapuche communities studied, taboos about fish consumption are little evident, but the scarce and careful use is prevalent in most of interviewees. For example, inhabitants refer to the way the extraction of any of the elements that compose the lakes and rivers should be based on the “*permission and reciprocity*” of the environment. For example, one informant told us…“*The waters give what they want to give and it’s thought that they often punish people, sending them illness or misfortune”*(Interviewee # 24) In all cases, the existence of strong “social control” over these environments is evident, with confirmed rules of usage, which imply punishment for those who infringe them, and which indirectly favour their conservation, since extractive activities are not promoted. Finally, since in the communities studied we found no environmental management practice in the literature or the discourses, we can assume that the high symbolic-religious value is what favours its preservation. In this sense, various versions of Mapuche myths tell of a great flood after which human beings, trying to save their lives in the face of rising waters, climbed the mountains to protect themselves. They could not hold on for long enough, however, and fell into the water, where they were transformed into fish [138, 141–144]. Although this story referring human reincarnation was not specifically told by interviewees, its content reveals the high symbolic value of consuming fish in this traditional culture.

### Tools and techniques used for fishing, past and present

According to informants, in past times traditional weapons were used, like the harpoon and the “*huachi”,* whereas nowadays even though they are still used by some, most people use modern tools based on hook and line (spinning, flyfishing, and tin and spoon), tools which currently predominate and are in common use among locals and sports fishermen in the region [4]. Of all the inhabitants interviewed, 50% mentioned using the harpoon and the *“huachi”,* and a lower percentage reported using modern tools. Both harpoon and *“huachi”* are constructed from a long stick of 1–2 m length, with a sharp element on one end: in the case of the harpoon, a knife, metal tip or piece of iron, and in the case of the *“huachi”,* generally a wire in the shape of a lasso. The preferred wood for the handle of these tools is caña colihue *Chusquea culeou, Bambuceae*. As indicated by its name in Mapudungun (“*coliu”,* cane and “*hue”,* place or abundance), this perennial species is abundant in the region, and its wood is hard, slippery, light, yet water resistant and does not rot easily, meaning that these tools can be kept and reused [145, 146]. Informants reported that the traditional tools were used in rivers and streams, requiring knowledge of the area and the habitat of the fish, and ability on the part of the fisherman in order to catch fish. Interviewees remembered past times when these tools were of common use in rivers during low water flowss (presumably during the Summer months of January to March), in environments with reduced water flow, or in still waters at the river edge. Also, during the time of year when some fishes swim up the rivers of the Cordillera, (presumably during the reproductive season), and are very abundant in rocky areas. They also told us how they would wait for a fish to appear or swam upriver to quickly skewer it with the harpoon. In the case of the *“huachi”,* the fishermen would try to wait till the fish swam inside the loop, and then tighten it firmly and lift it quickly out of the water, killing the fish with a blow.

With respect to the characteristics of modern tools, the interviewees mentioned that they enable them to continue fishing from the riverside while reaching further into the water bodies, and at the same time require less effort. These tools also make it possible for them to catch fish of other species and sizes. Records reveal the relationship that exists between environmental characteristics and the type of tool used for fishing. It could be said that in past times traditional tools were exclusively oriented towards subsistence needs and were in sporadic use, whereas the modern tools relate more to a recreational activity. During our interviews no specific details were recorded in terms of bait, boats or times of day for fishing, or the age of the fishermen, but interviewees did highlight the qualities attributed to “fishermen”; they should have experience of fishing in order to be successful. Finally, in terms of gender, according to our records, in these Patagonian environments fishing was and still is carried out by men, although a chance event may find both men and women fishing. In addition, fishing is an individual activity.

In our search for information, little mention was made by chroniclers of fishing among indigenous peoples in Patagonian aquatic environments. The identified, available sources coincide in indicating the use of harpoons in rivers, and the same methods currently used [144, 147]. Zapater [147] refers to fishing in the Imperial River (IX Region of La Araucanía, Chile) with colihue cane rods. *“In the months of February and March when they come upriver in greater numbers, the Indians fished like this, they stood one on each side of the river, a few rods’ distance from each other, armed with tapering solid rods, called coligue in their language, they put these rods in the water with a deadly thrust, they stab the fish which is densely packed in the water and by just stabbing it and pulling it out the fishermen catch in just a few hours enough to feed the inhabitants, which are many, from the neighbouring lands…”.* Furthermore, Coña [144] refers to fishing with pointed colihues (*Nothofagus dombeyi*), fish spears, tridents or harpoons, instruments which enable them to catch the fish in Boca Budi (IX Region of La Araucanía, Chile) at extremely low flow. With these methods of fishing the Mapuche in past times sustained their communities in times of famine.

### Changes in knowledge and fishing activities

In general, informants indicated that in the past, little or no fishing was carried out (92%), and that nowadays little fishing is done (97%). The various reasons given for the low incidence of fishing in the past were attributed to inhabitants: “*Our ancestors didn’t fish because they didn’t like it much and they didn’t need to because they had food, enough meat from large animals and the harvest*” (Interviewee # 14). “*They weren’t keen on fishing and were busy with the animals, with their animals they went out to catch “peludos”* (big hairy armadillos) (*Chaetophractus villosus*) (Interviewee # 6). Moreover, informants said that a great deal of experience was required, since “*only people or fishermen with experience could fish successfully*” (Interviewee # 22). As mentioned previously, inhabitants reported, “*the people didn’t use to fish out of respect for the lake*” (Interviewee # 24). One interviewee told us: “*The fish were not there to make money, nature is not for making money*”. “…*My grandparents spoke to me of the strength of the water, that everything had power, our nature has power, like the water, the lake, the mountains . The streams and springs also have power”* (Interviewee # 10) thus affirming the symbolic value and the feeling of respect we have previously spoken of.

In the past, fishing was seen as a difficult activity, and inefficient due to the considerable amount of time required to catch fish one by one. All the reasons given by informants are in synchrony with the ecological characteristics of aquatic environments in the region. In Patagonia the rivers and lakes present low abundance and diversity of fishes [89] in comparison, for example, with the highly productive environments of northern Argentina where there is greater abundance and species diversity [42]. For this reason the arrival of salmonids in these fluvial environments signifies not only an increase in the diversity and abundance of fishes but also in the probability of catching them. On the other hand, because the water bodies are poor in nutrients the Patagonian fishes have a low caloric value as compared to fish species in the north of the country, and in comparison with livestock.

According to inhabitants, fishing is currently carried out as an occasional activity and also for family or household consumption. For the families, the fish of the region constitute a complementary resource, allowing them to broaden or vary their diet. It could be said that among the Mapuche to the east of the cordillera, fishing was not in the past, nor is today, an important activity or one carried out frequently, in contrast with the Mapuche fishing groups on the Chilean side, which make liberal use of marine resources [148, 149].

Finally, our results coincide with ethnnohistorical sources where naturalists, chroniclers and travellers at the end of the 19th century and during the 20th century [64–68] make little reference to the relation between the Mapuche people and fluvial fishes. In the cases where mention is made of this, it applies to unusual, infrequent consumption of fish and a lack of knowledge about methods of capture on the part of the hunter-gatherer groups [57].

## Conclusions

The traditional ecological knowledge of the three Mapuche communities includes 5 species of fishes and 18 non-human beings (animals and/or sacred or mythological personages), guardians, protectors or owners of the fluvial environments. Although for the Mapuche people of the eastern side of the cordillera traditional fishing never represented a leading activity, but rather a secondary activity in response to dietary need, the water bodies in their surroundings are protected by customary norms which limit their use. We found a “reciprocal” relationship and “balance” in the studied communities between the people, the fish and the other beings that inhabit the waters, which is typical of a relational cosmovision, deeply connected with nature [120]. This emotional and intellectual positioning of the Mapuche communities is different from the current globalised market society where man and nature are generally dissociated. Traditional tales, “*epew*”, and ancients myths reflect this stance, revealing attitudes of preservation, respect and care, and also serve as oral vehicles that keep the memories alive. We confirmed that perceptions arise from the conceptions and beliefs of the Mapuche people which tend towards sustainability and conservation of continental aquatic ecosystems. The impact and interventions of man in these environments (e.g. the introduction of species) are experienced by the Mapuche as disturbing factors.

We can say that the body of traditional knowledge and its practical application are flexible in nature and adaptable to new socio-environmental situations. This is seen in the changes and incorporation of new fishing tools, ways of doing things and fishing techniques in response to changes in the environment and society. In Mapuche symbolism, exotic species such as trout, first constitute ill omens (“the white man has been close”), and later are incorporated in a sporadic manner into the diet of some individuals.

This paper contributes to the broader literature on freshwater resource management by providing empirical evidence of the critical role of local perceptions in promoting the sustainable management of natural resources. In addition, we consider that knowing and valuing the perceptions and symbolism of the indigenous peoples with regard to aquatic ecosystems constitutes an initial step in the integration of local traditional and scientific knowledge towards the preservation of Patagonian environments. It has been seen that the inclusion of ethnozoological knowledge in development projects constitutes a fundamental element in the analysis of concrete conservation problems and the management of natural resources [33].

The recovery of the word through this work has led to revitalization of this knowledge, and may therefore indicate a path towards the empowerment of these communities, legitimising their way of life and perceptions in the face of the notable transformations experienced in recent years due to the effects of population growth, development and globalisation. Many Mapuche families are interested in the restructuring of their productive activities, where sport fishing could occupy a place of interest. For this reason, recovery of this knowledge would strengthen the generation of intercultural projects which would not ignore Mapuche precepts and their significance in terms of use of the environment. Finally, we seek to draw attention to the need for integral studies which take cultural aspects (beliefs and perceptions) into account in the development of productive projects in our region.
